# Lymphedema in Endometrial Cancer Survivor: A Nationwide Cohort Study

**DOI:** 10.3390/jcm10204647

**Published:** 2021-10-11

**Authors:** Su-Jeong Lee, Jun-Pyo Myong, Yun-Hee Lee, Eui-Jin Cho, Sung-Jong Lee, Chan-Joo Kim, Jin-Hwi Kim

**Affiliations:** 1 Department of Obstetrics and Gynecology, Uijeongbu St. Mary’s Hospital, College of Medicine, The Catholic University of Korea, Seoul 06591, Korea; crystal_88@naver.com (S.-J.L.); twikkling@naver.com (E.-J.C.); chanjoo218@naver.com (C.-J.K.); 2 Department of Occupational & Environmental Medicine, Seoul St. Mary’s Hospital, College of Medicine, The Catholic University of Korea, Seoul 06591, Korea; medical001@catholic.ac.kr; 3 Department of Urology, Seoul St. Mary’s Hospital, College of Medicine, The Catholic University of Korea, Seoul 06591, Korea; eyh900@naver.com; 4 Department of Obstetrics and Gynecology, Seoul St. Mary’s Hospital, College of Medicine, The Catholic University of Korea, Seoul 06591, Korea; orlando@catholic.ac.kr

**Keywords:** endometrial cancer, lymphedema, sentinel lymph nodes, health care cost, Korea

## Abstract

Background: Endometrial cancer is the most common gynecological cancer in developed countries. Treatment-related lymphedema negatively affects the quality of life and function of patients. This study investigated the cumulative incidence and risk factors of, and utilization of health care resources for, lymphedema in patients with endometrial cancer. Methods: We conducted a nationwide, retrospective cohort study of women with endometrial cancer who underwent cancer-direct treatment using the Korean National Health Insurance Service (NHIS) database. Patients were categorized by age, region, income, and treatment modality. Cox proportional hazards regression models were used to analyze the incidence and risk factors of lymphedema. We also analyzed utilization of health care resources for lymphedema using diagnostic and treatment claim codes. Results: A total of 19,027 patients with endometrial cancer were evaluated between January 2004 and December 2017. Among them, 2493 (13.1%) developed lymphedema. Age (<40 years, adjusted odds ratio [aOR] = 1 vs. 40–59 years, aOR = 1.413; 95% confidence interval (CI) 1.203–1.66 vs. 60+ years, aOR = 1.472; 95% CI 1.239–1.748) and multimodal treatment (surgery only, aOR = 1 vs. surgery + radiation + chemotherapy, aOR = 2.571; 95% CI 2.27–2.912) are considered to be possible risk factors for lymphedema in patients with endometrial cancer (*p* < 0.001). The utilization of health care resources for the treatment of lymphedema has increased over the years. Conclusions: Lymphedema is a common complication affecting women with endometrial cancer and leads to an increase in national healthcare costs. Post-treatment surveillance of lymphedema, especially in high-risk groups, is needed.

## 1. Introduction

The prevalence of endometrial cancer is increasing among the gynecological cancers in developed countries [1]. In Korea, endometrial cancer ranked second in gynecological cancer prevalence in 2017, according to national cancer registration statistics [2].

Total hysterectomy and bilateral salpingo-oophorectomy with or without lymphadenectomy are the standard surgical treatments for endometrial cancer. Patient groups with poor prognostic factors such as high-grade histology, lymph-vascular invasion, parametrial invasion, and pelvic or para-aortic lymph node invasion need adjuvant radiation therapy and/or chemotherapy. In particular, when pelvic and para-aortic lymph node dissection or radiation therapy is combined with hysterectomy in an endometrial cancer patient, obstruction or compromise of the lymphatic system can occur, resulting in lymphedema [3,4].

Lymphedema presents symptoms such as pain and skin loss and induces activity restriction and psychosocial impairment. These can affect the patient’s compliance with treatment and life-long morbidity [5,6,7]. The incidence of lymphedema in endometrial cancer has been reported to range from 1.2% to 47% [3,4,8,9]. The prevalence of lymphedema in such a wide range may be due to differences in treatment methods for endometrial cancer, such as addition of adjuvant radiation therapy, or differences in the number of removed lymph nodes [4], but may be also due to unrecognized signs or symptoms of lymphedema. Obesity is a known risk factor of lymphedema in endometrial cancer, so that lymphedema may be masked in obese endometrial cancer patients [10]. Additionally, lymphedema in endometrial cancer often occurs bilaterally, while lymphedema in breast cancer patients typically occurs unilaterally [11]. This makes diagnosis of lymphedema in endometrial cancer patients difficult.

Standard treatment of lymphedema is complex decongestive therapy, which consists of manual lymphatic drainage, skin care, compression bands or stockings, and exercise. Physical therapy and pharmacological treatment, such as vitis vinifera extract and calcium dobesilate, are also widely used [12]. Lymphedema is a chronic condition and is often not curable; therefore, long-term socioeconomic burden is expected. There have been many studies around the world on the economic burden associated with treatment of lymphedema after surgery in other cancers like breast cancer [13,14,15]. However, there is a lack of research on the cost of treating lymphedema in endometrial cancer.

Thus, this study investigated the cumulative incidence and additional risk factors of, and utilization and cost of medical resources for lymphedema in patients with endometrial cancer by using the Korean National Health Insurance Service (NHIS). This will establish active counseling and provide appropriate treatment plans for endometrial cancer patients at risk of lymphedema.

## 2. Materials and Methods

### 2.1. Data Acquisition

South Korea has a universal health insurance program called the National Health Insurance Service (NHIS) that covers about 98% of the total Korean population [16,17]. The Korean Health Insurance Review and Assessment Service (HIRA) is a government institution that serves as an accurate assessment and quality evaluation system for the NHIS. Billing data for reimbursement of services provided by medical service providers to patients are evaluated and collected by HIRA; therefore, HIRA data are highly accurate for medical information. The HIRA database contains almost all inpatient and outpatient data from tertiary hospitals and community clinics, making a national population-based study available. This is a retrospective cohort study using full insurance data from HIRA between January 2004 to December 2017. For this study, we used the data with the approval of the HIRA data access committee, based on the Rules for Data Exploration and Utilization of the HIRA. To protect the privacy of patients, potentially identifiable personal information, including name, address, and date of birth, were not accessible. We also obtained Institutional Review Board (IRB) approval from the ethics committee of the Catholic University to use the data (approval number: UC17ZESI0053).

### 2.2. Study Population and Design

**Figure 1:**
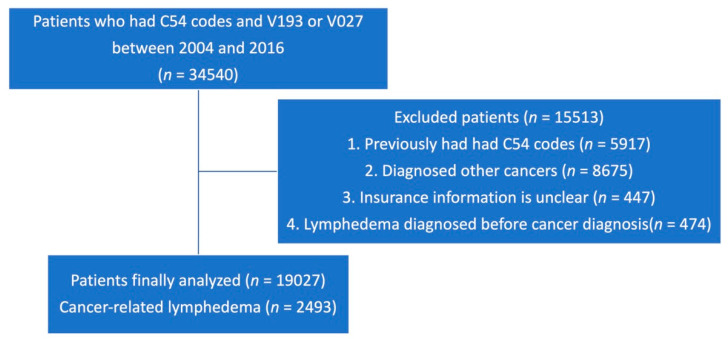
A schematic illustration of participant selection for the present study. A total of 34,540 patients with endometrial cancer were included in the analyses. Patients who were diagnosed before 2003, those with other primary cancers, or those with lymphedema diagnosed before cancer diagnosis were excluded.

To select the study group, we used the 10th revision of the International Statistical Classification of Diseases and Related Health Problems (ICD-10), the Health Insurance Medical Care Expenses (2017 and 2018 versions), and the HIRA Drug Ingredients Codes for diagnostic codes, surgery codes, and prescription codes. The study population, lymphedema patients who were diagnosed after endometrial cancer treatment, was defined as patients who had diagnostic codes for both endometrial cancer (ICD-10 code C54) and lymphedema (ICD-10 code I890) between 2004 to 2017. Among them, those who already had a lymphedema diagnosis prior to the first endometrial cancer diagnosis were excluded. Patients diagnosed with endometrial cancer from 2004 to 2005 were excluded for the wash-out period, so only newly diagnosed endometrial cancer patients were included (Figure 1).

The variables in this study include age at diagnosis, residence, income quartile, and endometrial cancer treatment modality. Age was categorized in three groups: <40 years old, 40–59 years old, and ≥60 years old. The residential area was classified into a metropolitan city group (urban areas such as Seoul, Busan, etc.) and a rural area group, using the area code of the medical institution. For income, based on the national health care insurance payment, the 20 classes were categorized into four stages. The claim codes of R4145 (total hysterectomy), HD059 (rotational irradiation—high energy), and KK151 (anticancer drug IV infusion) were used to determine the treatment method (surgery, radiation therapy, and chemotherapy, respectively) performed after the diagnosis of endometrial cancer. Radiation therapy and chemotherapy were also defined by using prescription codes. The analysis was divided into groups that received surgery, radiation therapy, or chemotherapy alone, and groups that combined these treatments. This study did not target specific cohorts but looked at the entire real-world data of all patients that actually occurred in Korea, so it is believed that there were a variety of treatments, including hormone therapy. In addition, it was thought that among them, those who did not receive special treatment, were not confirmed by insurance, or those who received treatment from overseas. These patient groups were classified into the “other” category.

Analysis of the pattern of utilization of lymphedema treatment was performed by using prescription codes. Medical treatment comprised remedies such as vitis vinifera extract (248902ATB) and calcium dobesilate (148101ATB). All medication codes for lymphedema were approved by HIRA. Claims for physical treatment included complex decongestive physical therapy (MM200) and pneumatic compression (MM190). The cost incurred for each treatment and drug was investigated.

### 2.3. Statistical Analysis

Fisher’s exact test and the Chi-square test were used to compare general characteristics of patients with endometrial cancer. Cumulative incidences of the primary variable were estimated by Kaplan–Meier survival curves and compared using log-rank tests. The incidence of lymphedema according to age, region, income, and treatment modalities was observed, and all results were estimated using Cox proportional hazards regression analysis. Two-tailed analyses were conducted, and statistical significance was considered at *p* < 0.05. Adjusted odd ratios (aORs) and 95% confidence intervals (CIs) were calculated. Statistical analyses were performed with SAS enterprise guide version 7.1 (SAS Institute Inc., Cary, NC, USA).

## 3. Results

### 3.1. Basal Characteristics

**Figure 2:**
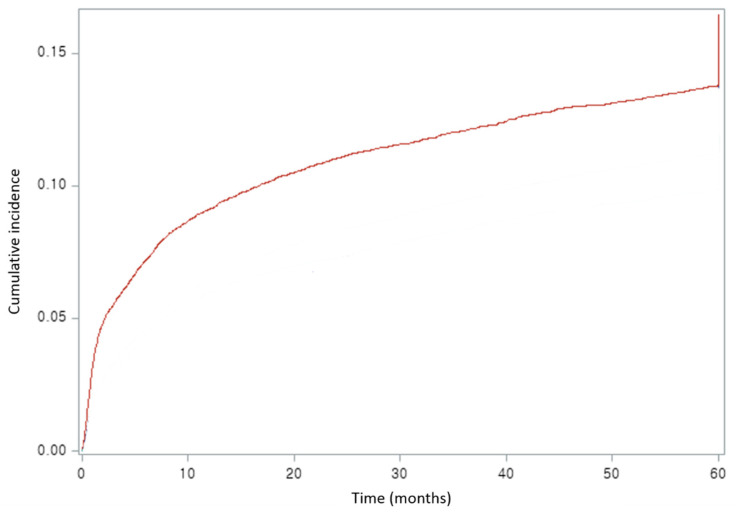
Cumulative incidence of lymphedema in endometrial cancer patients.

From January 2004 to December 2017, a total of 19,027 endometrial cancer patients were included, of which 2493 (13.1%) developed lymphedema (Figure 2). Table 1 shows the incidence of lymphedema in endometrial cancer according to the basal characteristics and treatment modality of the study group. About 88% of patients were diagnosed with endometrial cancer over the age of 40, and the incidence of endometrial cancer was the highest in the upper-quartile income group at 32.2%. Regarding residential area and treatment modality, 67.9% of people lived in urban areas, and about 95% of people were treated with surgery alone or a combination of surgery and other treatment modalities.

### 3.2. Risk Factors for Developing Lymphedema

**Figure 3:**
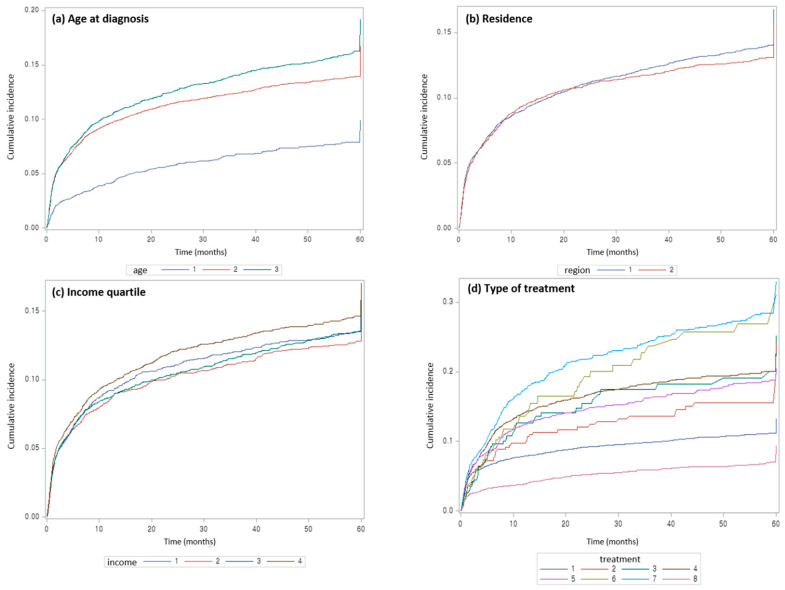
Cumulative incidence of lymphedema according to age at diagnosis, residence, income quartile, and endometrial cancer treatment modality. (**a**). Cumulative incidence of lymphedema according to age. (1; 0–39 years, 2; 40–59 years, 3; 60+ years old) (**b**). Cumulative incidence of lymphedema according to residence. (1; urban, 2; rural) (**c**). Cumulative incidence of lymphedema according to income. (1; lowest income, 2; lower, 3; upper, 4; highest) (**d**). Cumulative incidence of lymphedema according to treatment modalities. (1; Surgery, 2; Radiation, 3; Chemotherapy, 4; S + R, 5; S + C, 6; R + C, 7; S + R + C, 8; other).

Based on analysis using the Cox proportional hazards model, the cumulative incidence of lymphedema was high in patients with endometrial cancer who were over 40 years of age. When compared to the under-40 years age group, the hazard ratios (HR) for lymphedema were 1.41 (95% CI: 1.20–1.66, *p* < 0.0001) for those 40–59 years of age and 1.47 (95% CI: 1.24–1.75, *p* < 0.001) for the over-60 years age group (Table 1, Figure 3a). No significant difference in the incidence of lymphedema among patients with endometrial cancer was observed according to income and type of residential area (Table 1, Figure 3b,c).

According to the treatment modality, the incidence of lymphedema in endometrial cancer was significantly increased in the group that received radiotherapy alone (HR 1.42, 95% CI: 1.10–1.83, *p* = 0.0078) or chemotherapy alone (HR 1.81, 95% CI: 1.37–2.38, *p* < 0.0001), compared to the group that received surgery alone. As expected, the risk of lymphedema was significantly increased in the group that received multimodal treatment (i.e., two or more treatment modalities). In the group of patients who had received all of the surgery, radiation therapy, and chemotherapy, the incidence of lymphedema was the highest (HR 2.15, 95% CI: 1.65–2.78, *p* < 0.0001) (Table 1, Figure 3d).

### 3.3. Treatment Cost for Lymphedema in Endometrial Cancer

**Figure 4:**
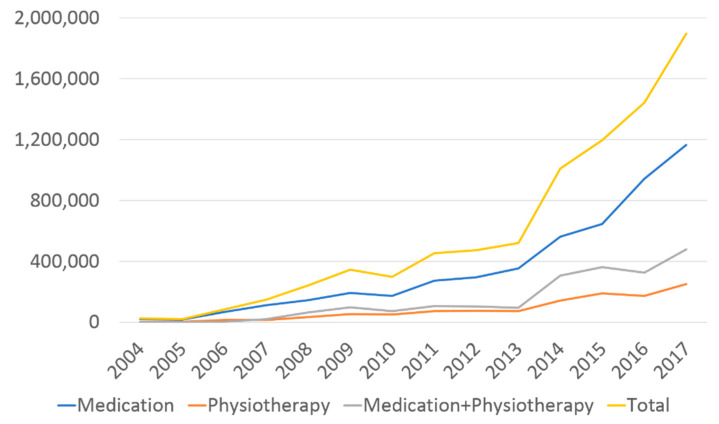
The trend of annual treatment cost for lymphedema in endometrial cancer.

According to the data from the National Health Insurance Service, the medical and physical therapy costs for lymphedema treatment in Korea from 2004 to 2017 are shown in Table 2. The total treatment cost for lymphedema increased from 26,039 USD in 2004 to 1,894,505 USD in 2017. The total cumulative treatment cost exceeded 8,155,093 USD in 2017 (Figure 4), and the cost of each treatment modality was also trending upwards. Among them, the cost of administering medical or physical therapy alone in 2017 had increased 56- and 57-fold, respectively, compared to 2004, while the cost for administering these two treatment regimens together increased about 500-fold.

## 4. Discussion

According to 2017 national cancer registration statistics, it is the second most common gynecological cancer, having increased approximately 5.3-fold compared to 1999. This is the most dramatic change when compared with a 4.6% decrease in cervical cancer and a 1.9% increase in ovarian cancer. In addition, the 10-year observed survival rate of endometrial cancer increased by 6.7% [2]. From this, the incidence and prevalence of endometrial cancer is expected to increase further in the future. For endometrial cancer survivors, lymphedema negatively affects physical and psychosocial functioning and quality of life [10]. Furthermore, the symptoms of lymphedema can be alleviated if treated, but it is not curable and remains a chronic condition. Failure to treat lymphedema leads to adverse clinical outcomes in endometrial cancer patients and increases the financial burden associated with the treatment of lymphedema. Therefore, the risk factors for lymphedema and the cost of treatment of lymphedema for endometrial cancer survivors are important issues that are worthy of study. At the same time, they are expected to serve as important factors in determining the future of health care policy.

In various studies, the incidence of lymphedema associated with endometrial cancer is widely reported, ranging from 1.2% in retrospective analyses [9] to 47% in prospective studies using patient surveys [4]. These differences may arise because treatment methods or follow-up periods differ from study to study. In addition, there were differences in the diagnosis of lymphedema depending on the study, such as using a subjective questionnaire completed by the patient or objective diagnostic criteria [18] observed by gynecologic oncologists. These inconsistencies contribute to difficulty in identifying the true rates of lymphedema. Many studies on the incidence and risk factors of lymphedema in endometrial cancer are mostly cohort studies based in a single center or in several centers. None of the existing Korean studies have performed population-based analyses of risk factors or treatment costs of lymphedema in endometrial cancer. This study investigated the incidence of lymphedema by analyzing cases of lymphedema in women diagnosed with endometrial cancer from 2004 to 2017 using Korean HIRA data, and the incidence was 13.1%.

Risk factors for developing lymphedema in endometrial cancer have been identified in many studies. The number of resected lymph nodes [19], presence of metastatic lymph nodes [10], old age, high body mass index (BMI), addition of adjuvant radiation therapy or chemotherapy, and decreased physical activity [3] are known risk factors for lymphedema in endometrial cancer. This study aims to investigate additional risk factors using a large-scale nationwide cohort database.

As expected, the incidence of lymphedema according to the endometrial cancer treatment modality increased when two or more treatment modalities were combined, compared to surgery alone. It may be interpreted that this is because adjuvant therapy is performed in patients with advanced endometrial cancer. In addition, the increase in the incidence of lymphedema observed in the elderly compared to patients with endometrial cancer under the age of 40 was also consistent with the results of previous studies. There was no difference in risk according to the patient’s income level and residential area. This is interpreted by the medical insurance system for cancer patients in Korea being well established, so that the amount to be borne by the patient for treatment is as small as 5% of the total cost, and the lower the income level, the greater the medical insurance benefits given. In addition, the treatment system related to endometrial cancer treatment is applied relatively consistently under the supervision of HIRA. Finally, tertiary centers are relatively evenly located throughout the country, which seems to be a factor that influences the good access to medical resources.

Lymphedema is often chronic and incurable, so treatment is aimed at alleviating the symptoms. Clear guidelines for lymphedema treatment are not established, and the drugs or therapies used for treatment are sometimes inconsistent. In this study, we analyzed the cost according to pharmacological and physical therapy prescription codes, which are used primarily to treat or relieve lymphedema symptoms. However, it was not possible to investigate the expenses paid by the patient outside the scope of medical insurance, such as those for compression stockings, massage therapy, and exercise. Additionally, in Korea, patients can easily access herbal medicine and acupuncture, which are generally considered expensive, but this burden cannot be known from the data available through the Insurance Corporation. In other words, the treatment-related costs presented in this study are likely to be underestimated. Nevertheless, the results of this study are significant, indicating that the burden of the cost of treating lymphedema increases every year.

The strength of this study was the large sample size based on the nationwide database. As mentioned earlier, NHIS covers about 98% of the total Korean population. Therefore, the sample size was sufficient to detect risk factors associated with developing lymphedema in endometrial cancer patients. It is meaningful to confirm the risk of lymphedema according to socioeconomic factors and residential areas, which are difficult to confirm in other studies using medical records.

The limitation of the data used in this study is lack of information about FIGO staging, pathological results, and lifestyle factors (e.g., smoking and obesity) because the NHIS does not provide these. This study provided information on the average cumulative incidence of lymphedema. However, endometrial cancer is generally diagnosed at an early stage in many cases, and it is known that lymphedema is more prevalent in advanced stage cases with lymph node metastasis. Therefore, it should be recognized that the incidence of lymphedema according to the stage of endometrial cancer may be different in clinical application.

In addition, it is difficult to know the specific method of surgery performed in the treatment of endometrial cancer. Considering that lymph node resection is a known risk factor for lymphedema in patients with endometrial cancer, whether lymph node resection was performed and how many lymph nodes were removed are important factors affecting the incidence of lymphedema. In particular, the recent trend of surgery for endometrial cancer is changing to reduce unnecessary lymph node dissections and further reduce the incidence of lymphedema by performing only sentinel lymph node biopsy in early-stage endometrial cancer [20]. As a new medical technology application prescription code for sentinel lymph node biopsy has been established, it is expected that incidence using big data can be reconfirmed in future study.

In the case of lymphedema, the investigation was possible only when the diagnosis code was entered. Therefore, the occurrence of lymphedema may be underestimated in cases where treatment is prescribed without a lymphedema diagnosis code, or in cases of mild lymphedema that does not require a prescription where the diagnosis code is omitted altogether.

This study is the first population-based study to identify incidence of lymphedema and risk factors associated with the occurrence of lymphedema in patients with endometrial cancer and is the first study to evaluate the use of medical resources for treatment-related burdens. Considering that the cost of treating lymphedema in patients with endometrial cancer is increasing year by year, active counseling and appropriate treatment plans for patients with risk factors for lymphedema will improve compliance and quality of life for endometrial cancer survivors.

## Figures and Tables

**Table 1:** Basal characteristics and risk factors of lymphedema.

	Total (n = 19,027)	Lymphedema			
		No (16,534, 86.9%)	Yes (2493, 13.1%)	HR (95% CI)	*p*-Value
Age					
<40	2276 (12.0%)	2133 (93.7%)	143 (6.3%)	1.00	
40–59	11,823 (62.1%)	10,273 (86.9%)	1550 (13.1%)	1.41 (1.20–1.66)	<0.0001
>60	4928 (25.9%)	4128 (83.8%)	800 (16.23%)	1.47 (1.24–1.75)	<0.0001
Income					
1~5	4720 (24.8%)	4117 (87.2%)	603 (12.8%)	0.94 (0.85–1.05)	0.2716
6~10	3653 (19.2%)	3214 (88.0%)	439 (12.0%)	0.88 (0.79–0.99)	0.0362
10~15	4533 (23.8%)	3950 (87.1%)	583 (12.9%)	0.93 (0.34–1.03)	0.1612
16~20	6121 (32.2%)	5253 (85.8%)	868 (14.2%)	1.00	
Residence					
Urban	12,919 (67.9%)	11,155 (86.4%)	1764 (14.2%)	1.00	
Rural	6108 (32.1%)	5379 (88.1%)	729 (11.9%)	0.95 (0.88–1.04)	0.2771
Treatment					
Surgery	8411 (44.2%)	7460 (88.7%)	951 (11.3%)	1.00	
Radiation	447 (2.4%)	384 (85.9%)	63 (14.1%)	1.42 (1.10–1.83)	0.0078
Chemotherapy	346 (1.8%)	293 (84.7%)	53 (15.3%)	1.81 (1.37–2.38)	<0.0001
S + R	2545 (13.4%)	2015 (79.2%)	530 (20.8%)	1.87 (1.68–2.08)	<0.0001
S + C	1404 (7.4%)	1171 (83.4%)	233 (16.6%)	1.61 (1.39–1.86)	<0.0001
R + C	319 (1.7%)	259 (81.2%)	60 (18.8%)	2.15 (1.65–2.78)	<0.0001
S + R + C	1314 (6.9%)	974 (74.1%)	340 (25.9%)	2.57 (2.27–2.91)	<0.0001
other	4241 (22.3%)	3978 (93.8%)	263 (6.2%)	0.68 (0.59–0.78)	<0.0001

S: surgery, R: radiation, C: chemotherapy.

**Table 2:** Annual treatment cost of lymphedema in endometrial cancer (unit: USD).

	Medication	Physiotherapy	Medication + Physiotherapy	Total
2004	20,696	4372	972	26,039
2005	16,313	2847	1495	20,654
2006	68,377	13,705	2890	84,972
2007	112,065	15,817	20,805	148,687
2008	144,772	34,270	63,320	242,362
2009	193,300	53,119	98,656	345,074
2010	172,975	50,082	74,014	297,070
2011	274,091	72,589	106,043	452,724
2012	293,853	75,010	104,135	472,998
2013	354,080	72,686	94,248	521,015
2014	560,701	142,837	305,295	1,008,833
2015	644,125	189,901	362,690	1,196,716
2016	943,558	173,691	326,195	1,443,444
2017	1,165,017	251,684	477,804	1,894,505

## Data Availability

Data are available upon request. All data relevant to the study are included in the article.
